# Influence of irrigation activation methods for intracanal medicament removal on the bond strength to dentin

**DOI:** 10.1186/s12903-026-09636-1

**Published:** 2026-08-15

**Authors:** Doğancan Sevgen, Şeref Nur Mutlu

**Affiliations:** 1 Department of Endodontics, Faculty of Dentistry, Necmettin Erbakan University, Konya, Türkiye; 2 Vocational School of Health Services, Necmettin Erbakan University, Konya, 42090 Türkiye

**Keywords:** Bond strength, Bio-C Temp, AH Plus, Calcium hydroxide, Ultrasonic activation, Bioceramics

## Abstract

**Background:**

The aim of this study was to comparatively evaluate the effects of removing calcium silicate–based (Bio-C Temp) and calcium hydroxide–based (UltraCal XS) intracanal medicaments via manual and ultrasonic activation methods on the bond strength of bioceramic-based (Bioserra) and resin-based (AH Plus) root canal sealers to dentin.

**Methods:**

Eighty single-rooted, single-canal human teeth were used in this study. After root canal preparation, the samples were randomly assigned to three main groups: UltraCal XS (*n* = 32), Bio-C Temp (*n* = 32), and a control group with no medicament application (*n* = 16). The medicament groups were further subdivided according to the removal technique into manual irrigation and ultrasonic activation subgroups. The root canals were obturated via either AH Plus or Bioserra sealers. Push-out bond strength tests were performed on the obtained sections, and failure modes were evaluated under a stereomicroscope.

**Results:**

Three-way ANOVA revealed that only the medicament type significantly affected the bond strength (*p* = 0.001); the activation method and sealer type did not (*p* > 0.05). In the UltraCal XS groups, where the medicament was removed via manual irrigation, the bond strength values were significantly lower than those in the Bio-C Temp and control groups (*p* < 0.05). Compared with manual irrigation, ultrasonic activation significantly increased the bond strength of the AH Plus sealer in the UltraCal XS group (*p* < 0.05). However, no statistically significant differences were detected between activation methods (manual or ultrasonic) or sealer types (AH Plus or Bioserra) in the Bio-C Temp groups. Mixed-type failure was the most frequently observed failure mode.

**Conclusions:**

The type of intracanal medicament was the only factor with a significant overall effect on bond strength. Manual irrigation appeared to reduce the bond strength with calcium hydroxide–based UltraCal XS, whereas ultrasonic activation showed no clear advantage for calcium silicate–based Bio-C Temp.

## Introduction

Intracanal medicaments aim to reduce the microbial load by diffusing into areas of the root canal system that are inaccessible to mechanical instruments [1]. For this purpose, calcium hydroxide–based preparations have been widely used for many years [2]. Calcium hydroxide exhibits antibacterial activity due to its high pH and promotes hard tissue formation [3]. Currently, ready-to-use calcium hydroxide pastes with added radiopacity are frequently preferred in clinical practice [4]. In recent years, advances in dental material technology have led to the introduction of new-generation calcium silicate–based medicaments with bioactive properties and high alkaline pH values [5, 6]. Bio-C Temp is a contemporary example of this group and has attracted attention because of its biocompatibility and antimicrobial effectiveness [4, 7].

Despite the antimicrobial benefits provided by intracanal medicaments, their complete removal from the root canal prior to obturation is critical for the success of the subsequent treatment stages [8, 9]. Residual medicament remnants on dentinal walls may hinder the penetration of sealers into dentinal tubules and impair the adaptation of the filling material, thereby compromising sealing quality [10, 11]. However, conventional syringe-and-needle irrigation techniques are known to be insufficient for completely removing medicaments, especially in complex canal anatomies and the apical third [12, 13]. Therefore, various activation techniques, including sonic, ultrasonic, and laser-assisted methods, have been recommended to increase the effectiveness of irrigation solutions and improve the removal of medicament residues [4, 5].

In addition to disinfection, hermetic obturation of the root canal system is essential for the success of endodontic treatment [14]. At this stage, epoxy resin–based sealers such as AH Plus, considered the gold standard, as well as bioceramic-based sealers with biocompatible and osteoinductive properties (e.g., Bioserra), have gained popularity in contemporary practice [15]. The influence of the intracanal medicament type and removal technique on the bond strength of these different sealer types to dentin is a clinically relevant issue that requires further clarification.

Therefore, the aim of this study was to comparatively evaluate the effects of removing calcium silicate–based (Bio-C Temp) and calcium hydroxide–based (UltraCal XS) intracanal medicaments via different irrigation techniques on the bond strength of bioceramic-based (Bioserra) and resin-based (AH Plus) root canal sealers to dentin.

## Materials and methods

### Sample selection and preparation

This in vitro study was approved by the Ethics Committee of the Faculty of Dentistry, Necmettin Erbakan University (Approval No: 2025/581). Eighty single-rooted, single-canal mandibular premolar and canine teeth extracted for periodontal or prosthetic reasons, free of caries, cracks, or fractures, and with fully developed roots were included. Following cleaning of the root surfaces, the teeth were stored in 1% thymol solution to prevent microbial degradation. To ensure standardization, all the samples were examined under a stereomicroscope (Olympus SZ-40) at ×12 magnification, and periapical radiographs were taken to confirm the presence of a single canal. The crowns were sectioned via a low-speed diamond saw (IsoMet, Buehler, IL, USA) to obtain a standardized root length of 12 mm.

### Root canal preparation

Root canals were prepared via the crown-down technique with the EndoArt Gold rotary system (İnci Dental, Istanbul, Türkiye) to an apical size of ISO #30 with a 0.04 taper. During instrumentation, canals were irrigated with 2 mL of 5.25% NaOCl after each file change (total volume: 10 mL).

### Experimental group allocation

The samples were divided into three main groups according to the type of intracanal medicament used: Group I (*n* = 32) consisted of samples treated with calcium hydroxide–based UltraCal XS; Group II (*n* = 32) consisted of samples treated with calcium silicate-based Bio-C Temp; and Group III (control, *n* = 16) consisted of samples in which no intracanal medicament was applied.

The samples in Groups I and II were further subdivided into two subgroups based on the irrigation activation method used during medicament removal (a: manual irrigation, b: ultrasonic activation).

### Post-instrumentation ultrasonic activation

Following the completion of instrumentation, canals were irrigated with 5.25% NaOCl to remove residual debris. In the ultrasonic subgroups (Ib and IIb), this NaOCl irrigation step was activated for 20 s via a Woodpecker Endo 3 ultrasonic device (Guilin Woodpecker Medical Instrument Co., Ltd., China) at power setting E2 with an ED96 tip positioned 1 mm short of working length. In the manual subgroups (Ia and IIa), the same volume of NaOCl was delivered via syringe irrigation without ultrasonic activation. Following this step, smear layer removal was performed with 5 mL of 17% EDTA, followed by a final rinse with 5 mL of distilled water, which was identical in all subgroups.

### Intracanal medicament application

After the intracanal medicaments were placed according to the manufacturers’ instructions, the canal orifices were sealed with temporary filling material, and all the samples were incubated at 37 °C in 100% humidity for one week.

### Removal of intracanal medicaments

After the incubation period, the intracanal medicaments were removed via sequential irrigation with 5 mL of 5.25% NaOCl, 5 mL of 17% EDTA, and 5 mL of distilled water. In the passive ultrasonic activation subgroups (Ib and IIb), the EDTA irrigation step was activated for 20 seconds via the same ultrasonic device and settings described above. The irrigant volumes were identical to those used in the manual subgroups.”

### Root canal observation

For obturation, each main group was further subdivided according to the sealer used:

(1) AH Plus or (2) Bioserra. All canals were obturated via the single-cone technique with ISO #30 and 0.04 taper gutta-percha cones (EndoArt, İnci Dental, Istanbul, Türkiye). AH Plus was placed using a lentulo spiral, while Bioserra was applied via its proprietary syringe system.

### Specimen sectioning

After obturation, the samples were embedded in cold-curing acrylic resin. Horizontal Sect.  2 mm in thickness were obtained from the middle third of the root (6 mm from the coronal surface) via a low-speed cutting device (IsoMet, Buehler, IL, USA). The thickness of each section was verified via a digital caliper.

### Push-out bond strength test

For the push-out test, the samples were positioned with the apical surface facing upward in a universal testing machine (BESMAK A.Ş., Ankara, Türkiye). Load was applied in an apical-to-coronal direction at a crosshead speed of 1 mm/min using pluggers (0.5, 0.7, or 0.9 mm) corresponding to the canal diameter until bond failure occurred. The bond strength values were calculated in megapascals (MPa) by dividing the maximum force (N) by the bonded surface area (A). The bonded surface area (A) of each section was calculated via the following formula for the lateral surface area of a truncated cone: A = π (R + r) √ [(R − r) ² + h²], where R and r represent the coronal and apical radii of the root canal, respectively, and h represents the thickness of the Sect [16]. The coronal and apical canal diameters, as well as the thickness of each section, were measured with a digital caliper (Yu-Su, China).

### Failure mode analysis

After testing, all the sections were examined under a stereomicroscope (Olympus SZ-40, Japan) at ×40 magnification to determine failure modes such as adhesive, cohesive, or mixed [17].

### Statistical analysis

Statistical analysis was performed via SPSS version 27.0. Three-way analysis of variance (ANOVA) was conducted with intracanal medicament (UltraCal XS, Bio-C Temp, control), the irrigation activation method (manual, ultrasonic), and the root canal sealer (AH Plus, Bioserra) as independent factors to evaluate main effects and their interactions on push‒out bond strength. Levene’s test was used to verify the homogeneity of variances. For descriptive purposes, additional pairwise comparisons within specific medicament–sealer–activation combinations were performed via one-way ANOVA and independent-samples t tests, with Bonferroni correction applied to identify the source of significant differences. The level of statistical significance was set at *p* < 0.05 for all analyses.

## Results

### Three-way ANOVA: main effects and interactions

Three-way ANOVA was performed to evaluate the main effects of intracanal medicament type, irrigation activation method, and root canal sealer, as well as their interactions, on push‒out bond strength (Table 1). A statistically significant main effect was found for intracanal medicament type (F = 10.14, *p* = 0.001, partial η² = 0.225), whereas the irrigation activation method (F = 0.13, *p* = 0.724, partial η² = 0.002) and the root canal sealer (F = 0.64, *p* = 0.425, partial η² = 0.009) did not have statistically significant main effects. None of the two-way interactions (medicament × activation method, *p* = 0.122; medicament × sealer, *p* = 0.358; activation method × sealer, *p* = 0.430) or the three-way interaction (*p* = 0.877) reached statistical significance. The overall model explained approximately 15% of the variance in the bond strength (adjusted R² = 0.150). Levene’s test confirmed homogeneity of variances (*p* > 0.05). The data obtained from statistical analyses and stereomicroscopic examinations are presented below.

**Table 1:** Three-way ANOVA results for the push–out bond strength

Source	Type III Sum of Squares	df	Mean Square	F	*p*	Partial η²	Observed Power
Corrected Model	187,921	9	20,880	2,814	0,007*	0,266	0,939
Intercept	1363,627	1	1363,627	183,757	0,001*	0,724	1,000
Medicament	150,446	2	75,223	10,137	0,001*	0,225	0,983
Activation Method	0,935	1	0,935	0,126	0,724	0,002	0,064
Sealer	4,776	1	4,776	0,644	0,425	0,009	0,124
Medicament × Activation Method	18,171	1	18,171	2,449	0,122	0,034	0,339
Medicament × Sealer	15,474	1	7,737	1,043	0,358	0,029	0,225
Activation Method × Sealer	4,666	2	4,666	0,629	0,430	0,009	0,122
Medicament × Activation Method × Sealer	0,179	1	0,179	0,024	0,877	0,000	0,053
Error	519,458	70	7,421				
Total	2100,902	80					
Corrected Total	707,379	79					

*p < 0,05, Adjusted R^2^ = 0,150

For descriptive purposes, pairwise comparisons within specific medicament–sealer–activation combinations are presented below (Tables 2, 3 and 4); however, as the omnibus three-way ANOVA did not identify significant interaction effects, these comparisons should be interpreted as exploratory and hypothesis-generating rather than confirmatory.

**Table 2:** Comparison of root canal sealer bond strength using the same intracanal medicament

	Sealer	N	Mean	S.D.	t	*p*
Ultracal XS + Manual	AH Plus	8	1,93	1,06	0,157	0,878
	Bioserra	8	1,80	1,92		
Ultracal XS+ Ultrasonic	AH Plus	8	3,88	2,11	0,857	0,635
	Bioserra	8	2,47	1,70		
Bio-C Temp + Manual	AH Plus	8	5,38	4,23	1,475	0,162
	Bioserra	8	6,39	4,09		
Bio-C Temp + Ultrasonic	AH Plus	8	4,99	2,04	-0,119	0,907
	Bioserra	8	5,13	2,65		
Control	AH Plus	8	5,54	2,74	0,923	0,372
	Bioserra	8	4,22	2,96		

*p < 0,05

**Table 3:** Comparison of medicament effects on bond strength under identical sealer and activation conditions

Activation Method	Sealer	Medicament	*N*	Mean	S.D.	t	*p*
Manual Activation	AH Plus	Kontrol	8	5,54	2,74	3,771	0,040*
		Ultracal XS	8	1,93	1,06		
		Bio-C Temp	8	5,38	4,23		
	Bioserra	Kontrol	8	4,22	2,96	4,335	0,027*
		Ultracal XS	8	1,80	1,92		
		Bio-C Temp	8	6,39	4,09		
Ultrasonic Activation	AH Plus	Ultracal XS	8	3,88	2,11	1,146	0,303
		Bio-C Temp	8	4,99	2,04		
	Bioserra	Ultracal XS	8	2,47	1,70	5,736	0,003*
		Bio-C Temp	8	5,13	2,65		

*p < 0.05

**Table 4:** Effect of Activation Methods on Bond Strength with the Same Intracanal Medicament and Sealer

Medicament-Sealer	Activation method	*N*	Mean.	S.D.	t	*p*
Ultracal XS + AH Plus	Manual Activation	8	1,93	1,06	-2,338	0,035*
	Ultrasonic Activation	8	3,88	2,11		
Ultracal XS + Bioserra	Manual Activation	8	1,80	1,92	-0,730	0,477
	Ultrasonic Activation	8	2,47	1,70		
Bio-C Temp + AH Plus	Manual Activation	8	5,38	4,23	0,235	0,818
	Ultrasonic Activation	8	4,99	2,04		
Bio-C Temp + Bioserra	Manual Activation	8	6,39	4,09	0,731	0,477
	Ultrasonic Activation	8	5,13	2,65		

*p < 0,05

### Comparison of root canal sealers according to bond strength

In the group in which UltraCal XS was removed via ultrasonic activation, no statistically significant difference was found in the bond strength between AH Plus and Bioserra sealers (*p* > 0.05). Similarly, in the groups where UltraCal XS was removed by manual irrigation, as well as in all the Bio-C Temp groups regardless of the activation method (manual or ultrasonic), no statistically significant difference was observed between the two sealers in terms of bond strength (*p* > 0.05). In the control group, no significant difference in bond strength was detected between the AH Plus and Bioserra sealers (Table 2).

### Effect of intracanal medicaments on bond strength

When the effects of intracanal medicaments were evaluated under the same sealer and activation conditions, significant differences were observed in the manually activated groups. Among the samples obtained with both AH Plus and Bioserra, the UltraCal XS group, in which the medicament was removed via manual activation, presented significantly lower bond strength values than the Bio-C Temp and control groups did (*p* < 0.05). According to the tabulated data, the highest bond strength values under manual activation conditions were observed in the Bio-C Temp groups. In the ultrasonically activated groups, no statistically significant difference was found between the medicaments when AH Plus was used as the sealer (*p* > 0.05). However, in the Bioserra groups, Bio-C Temp resulted in significantly greater bond strength values than UltraCal XS did under ultrasonic activation (*p* < 0.05) (Table 3).

### Effects of irrigation activation methods on bond strength

When the effect of the activation method (manual or ultrasonic) was evaluated within the same intracanal medicament and root canal sealer combinations, a statistically significant difference was observed in the group in which UltraCal XS and AH Plus were used together. Compared with those subjected to manual activation, the samples subjected to ultrasonic activation presented significantly greater bond strength (*p* < 0.05). For the other combinations (UltraCal XS + Bioserra and all Bio-C Temp groups), no statistically significant difference was detected between the activation methods (*p* > 0.05). However, numerical analysis revealed that in the Bio-C Temp groups, manual activation yielded higher mean bond strength values than ultrasonic activation did (Table 4). This finding is discussed further below.

### Evaluation of failure modes

**Fig. 1:**
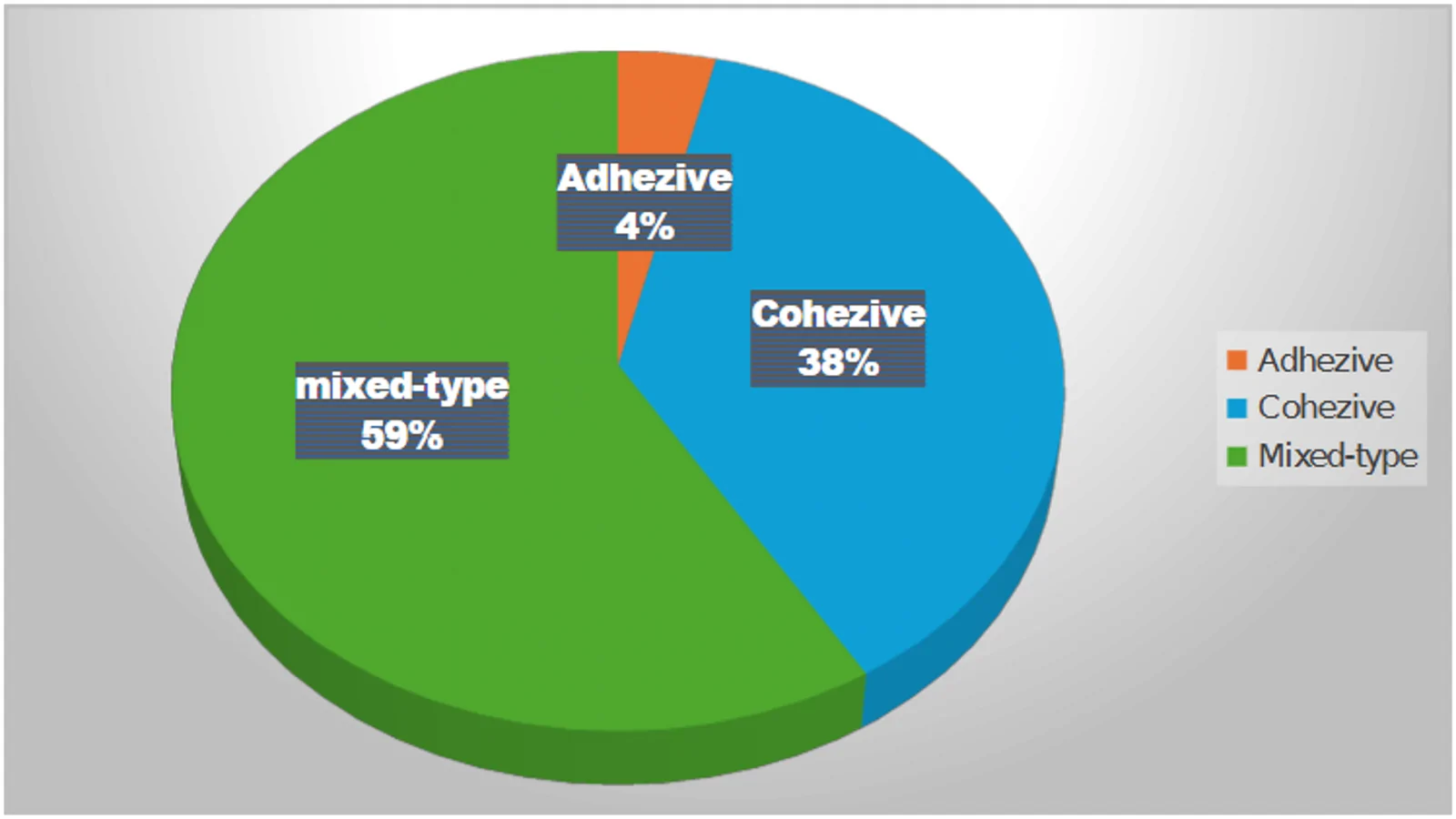
Distribution of failure modes across all groups

Stereomicroscopic analysis revealed that the most frequently observed failure mode across all the groups was mixed-type failure. In particular, the mixed failure rate reached 75% in groups such as UltraCal XS–Manual–AH Plus and UltraCal XS–Ultrasonic–AH Plus. Adhesive failure was the least frequently observed failure mode and was predominantly detected in the control group (12.5%). In contrast, cohesive failure was mostly observed in the UltraCal XS–Ultrasonic–Bioserra and Bio-C Temp–Manual–Bioserra groups, with a rate of 62.5% (Fig. 1).

## Discussion

Three-way ANOVA revealed a highly significant main effect of intracanal medicament type on push-out bond strength (*p* = 0.001), whereas neither the irrigation activation method nor the root canal sealer demonstrated significant main effects. Furthermore, no significant interactions were detected among the three variables. This pronounced main effect indicates that the application and specific type of interappointment dressing serve as the primary determinants of subsequent bonding efficacy, superseding the influence of sealer formulation or the activation technique. This conclusion is corroborated by several primary in vitro studies demonstrating that residual calcium hydroxide consistently compromises the push-out bond strength of various endodontic sealers, including epoxy resin, MTA, and bioceramic-based formulations [17–19]. Additionally, a recent study by Almohareb et al. revealed that prior application of Bio-C Temp, but not calcium hydroxide, significantly reduced the dentinal bond strength of bioceramic root canal sealers [20]. Collectively, these findings validate our analytical model: the adverse impact of medicament remnants acts as an independent, overriding factor that cannot be readily mitigated by adopting advanced sealer technologies or specific irrigation activation protocols. Given the lack of statistically significant interactions among the evaluated factors, the subsequent subgroup evaluations derived from the pairwise comparisons (Tables 2, 3 and 4) should be interpreted as exploratory and hypothesis-generating rather than indicative of confirmed interaction effects.

In contrast, AH Plus did not significantly differ in terms of medicament type, possibly reflecting its micromechanical interlocking capacity and chemical bonding with dentinal collagen [21, 22], although the effect of calcium hydroxide on resin-based sealer bond strength remains inconsistent across studies, ranging from neutral to positive to negative [23, 24].

In the pairwise comparison, ultrasonic activation was associated with a significant increase in AH Plus bond strength within the UltraCal XS group, which is consistent with the acoustic streaming and cavitation effects generally attributed to ultrasonic energy [25, 26]; a corresponding increase was not observed for Bioserra, which may point to differences in surface chemistry between sealer types, although this was not directly tested.

In the Bio-C Temp groups, no significant difference was found between AH Plus and Bioserra regardless of the activation method, although Bioserra presented numerically higher values, potentially related to the shared calcium silicate–based composition of Bio-C Temp and Bioserra [27, 28]; this numerical trend did not reach significance and warrants cautious interpretation. In the pairwise comparison within the Bioserra–ultrasonic subgroup, Bio-C Temp resulted in significantly greater bond strength than UltraCal XS [27, 28].

The lack of a significant benefit of ultrasonic activation over manual irrigation in the Bio-C Temp groups may, at least in part, reflect the reportedly strong affinity of this calcium silicate-based intracanal medicament for dentin and the inherent difficulty associated with its complete removal, although this interpretation remains speculative. Microcomputed tomography analyses have shown that no currently available technique, including passive ultrasonic irrigation, achieves complete removal of Bio-C Temp from the root canal system [29, 30], which may be related to its polyethylene glycol–based vehicle, which is used to maintain paste consistency and prevent hardening [20]. This duration- and protocol-dependent cleaning efficacy was further highlighted by Só et al., who demonstrated that even extended, cumulative activation protocols (three 20-second cycles) fail to fully eliminate Bio-C Temp residues from the root canal anatomy [30].

### Limitations

This study was conducted under in vitro conditions and therefore does not fully replicate the dynamic environment of the clinical setting. Additionally, the study was limited by the restricted number of intracanal medicaments, a single irrigation protocol, and one ultrasonic activation duration. The relatively small sample size per subgroup (*n* = 8) may have limited the statistical power to detect differences, particularly for numerical trends that did not reach statistical significance; future studies with larger sample sizes (e.g., *n* = 12–15) are recommended. Furthermore, the use of single-rooted, single-canal teeth, while advantageous for standardization, does not reflect the anatomical complexity of molar teeth, including isthmuses and lateral canals. The one-week incubation period may also not fully represent clinical scenarios in which intracanal medicaments are left in place for longer durations, potentially resulting in deeper carbonation of calcium hydroxide or more extensive biomineralization of calcium silicate–based materials. For calcium silicate-based intracanal medicaments such as Bio-C Temp, which are difficult to remove, further studies evaluating longer ultrasonic activation times and alternative irrigation techniques are warranted.

## Conclusions

Within the limitations of this study, intracanal medicament type had a significant overall effect on push-out bond strength, whereas the irrigation activation method and root canal sealer did not have significant main effects when all the factors were considered together. Nonetheless, exploratory cell-specific comparisons suggested that ultrasonic activation may improve bond strength in canals medicated with calcium hydroxide–based materials under specific sealer conditions, whereas its benefit appeared limited in the calcium silicate–based medicament groups. These findings indicate that medicament selection should be carefully considered to optimize the bonding performance of root canal sealers, while the role of the activation protocol warrants further investigation with larger sample sizes.

## Data Availability

The datasets used and analyzed during the current study are available from the corresponding author on reasonable request.

## Abbreviations

ANOVA: Analysis of variance
EDTA: Ethylenediaminetetraacetic acid
ISO: International Organization for Standardization
MPa: Megapascal
N: Newton
NaOCl: Sodium hypochlorite
PUI: Passive ultrasonic irrigation
SPSS: Statistical Package for Social Sciences
